# Development of Biocompatible, UV and NIR Excitable Nanoparticles with Multiwavelength Emission and Enhanced Colloidal Stability

**DOI:** 10.1021/acsmaterialsau.4c00151

**Published:** 2025-01-01

**Authors:** Egle Ezerskyte, Greta Butkiene, Arturas Katelnikovas, Vaidas Klimkevicius

**Affiliations:** † Institute of Chemistry, Faculty of Chemistry and Geosciences, Vilnius University, Naugarduko 24, LT-03225 Vilnius, Lithuania; ‡ Biomedical Physics Laboratory, National Cancer Institute, Baublio 3b, LT-08406 Vilnius, Lithuania

**Keywords:** nontoxic, luminescence, upconversion, core−shell−shell, NaGdF_4_, lanthanides

## Abstract

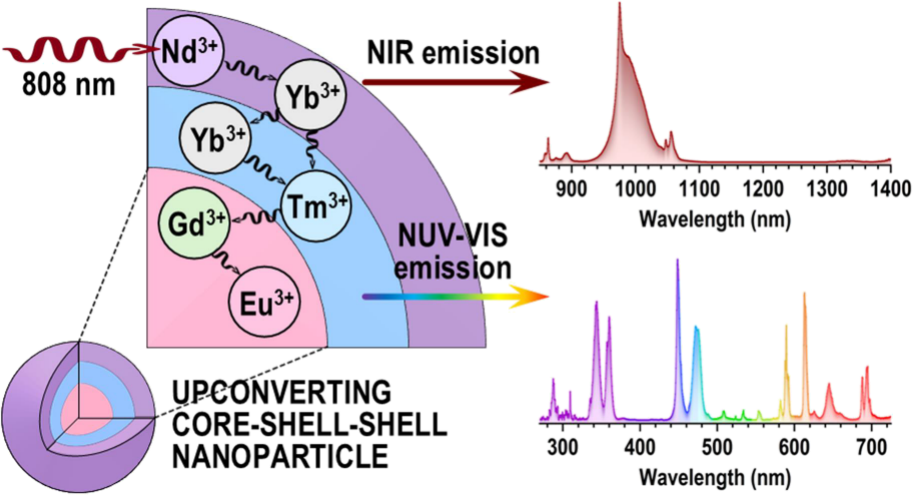

The development of functional nanoprobes for biomedical applications is highly important in the field of modern nanotechnology. Due to strict requirements, such as the ability to be excited using irradiation, which allows deep tissue penetration, nonblinking behavior, and good optical and colloidal stability, the choice of nanoparticles is limited, and their synthesis is challenging. Among all of the functional nanoprobes for biomedical purposes, upconverting nanoparticles, especially those with more complex architectures (e.g., core–shell or core–shell–shell), are the most promising candidates. This study demonstrates advanced synthetic routes for constructing biocompatible nanoprobes with tunable optical properties and colloidal stability. The core–shell–shell architecture of the nanoprobes allows excitation from at least four sources, such as 272 and 394 nm of near-ultraviolet (near-UV) irradiation and 980 and 808 nm near-infrared (NIR) lasers. Furthermore, Gd-matrix-based nanoprobes doped with lanthanide ions (Nd^3+^, Yb^3+^, Tm^3+^, and Eu^3+^) are known for their paramagnetic properties for magnetic resonance imaging (MRI) imaging as well as upconversion luminescence with diverse emission bands across the entire visible spectrum. This feature is highly desirable for photodynamic therapy applications, as the upconversion emission of the proposed nanoprobes could overlap with the absorption band of commonly used photosensitizers and could potentially result in an efficient energy transfer process and enhanced generation of reactive oxygen species or singlet oxygen.

## Introduction

Recent advancements in nanotechnology have spurred the development of innovative materials designed for biomedical applications.^1−3^ Among these, upconverting luminescent nanoparticles (UCNPs) are of particular interest due to their numerous advantages. First, modern methods allow the synthesis of inorganic UCNPs with precise control of their size, shape, and crystal structure, all of which can be tailored to suit the intended use.^4−6^ In biomedical applications, the small size of UCNPs facilitates their migration through cells via endocytosis, while their negligible solubility and physicochemical stability ensure that no potentially toxic ions or decomposition products are released.^7−10^ Second, upconverting materials can combine and convert two or more low-energy photons (usually NIR) to higher-energy radiation (visible (VIS) or even ultraviolet (UV)), offering several benefits.^11^ Biological tissues have optical transparency windows in the NIR region, known as the first biological window (NIR-I window, approximately 700–1000 nm) and the second biological window (NIR-II window, approximately 1000–1700 nm), where both optical absorption and scattering are considerably diminished due to lower water absorbance (by at least 90%).^12−15^ Therefore, the ability to excite UCNPs with 980 or 808 nm laser radiation is favorable, considering their use in biophotonics. However, although most research papers describe employing 980 nm laser irradiation for upconversion, 808 nm laser radiation is more advantageous from the perspective of optical transparency. While both wavelengths fall within the first biological window, studies have shown that 980 nm irradiation results in greater absorption of water molecules compared to 808 nm irradiation, leading to harmful thermal effects in tissues.^12^ Consequently, 808 nm irradiation allows for deeper tissue penetration. Shifting from the commonly used 980 nm irradiation to the more advantageous 808 nm requires Nd^3+^ ions to be present in the structure of UCNPs (because incorporating only Yb^3+^ ions as sensitizers enables efficient UCNP excitation only with 980 nm irradiation).^16^ It is also important to note that the absorption cross-section of Nd^3+^ ions for the 808 nm wavelength is up to 1 order of magnitude higher than that of Yb^3+^ ions for the 980 nm wavelength.^17^ However, if both sensitizers are present in the chemical composition of UCNPs, they offer the flexibility to choose between 980 and 808 nm excitation based on specific circumstances. In addition, both Yb^3+^ and Nd^3+^ ions exhibit emission in the second biological window, enabling protein autofluorescence-unaffected optical imaging using an NIR camera.^18^ Most importantly, the combination of Yb^3+^ and Nd^3+^ with other lanthanides (e.g., Er^3+^, Tm^3+^, Eu^3+^, and so forth) presents extensive opportunities for manipulating the emission spectra of UCNPs across the UV to VIS range, suggesting numerous potential applications in biosciences.^19−21^ For instance, one of the most extensively studied upconverting systems is the Yb^3+^ and Er^3+^ pair. Typically, the highest intensity of Er^3+^ emission in UCNPs is observed in the green (^4^S_3/2_ → ^4^I_15/2_ optical transition) and red (^4^F_9/2_ → ^4^I_15/2_ optical transition) ranges of the visible spectrum, making these UCNPs widely reported as promising nanoprobes for bioimaging or for activation of photosensitizers that generate reactive oxygen species (ROS) during photodynamic therapy (PDT).^22,23^ Additionally, the Er^3+^ emission in the green spectral region is distinctive due to its two thermally coupled energy levels, ^2^H_11/2_ and ^4^S_3/2_, with an energy difference of approximately 700–800 cm^–1^.^24^ This characteristic enables Er^3+^-doped nanomaterials to function as localized temperature sensors in various cells and tissues.^19,25^ Another popular upconverting ion pair is Yb^3+^ and Tm^3+^, with Tm^3+^ being notable for its intense emission lines in the UV and blue spectral ranges (optical transitions in the UV range: ^1^I_6_ → ^3^H_6_ (ca. 288 nm), ^1^I_6_ → ^3^F_4_ (ca. 343 nm), and ^1^D_2_ → ^3^H_6_ (ca. 360 nm); optical transitions in the blue range: ^1^D_2_ → ^3^F_4_ (ca. 448 nm) and ^1^G_4_ → ^3^H_6_ (ca. 473 nm)).^26^ UCNPs doped with this sensitizer-activator ion pair could potentially be used for the targeted release of photoactive drugs that are attached to the surface of UCNPs.^27−29^ The luminescence of Tm^3+^ can also be utilized in bioimaging with an NIR camera, as these ions emit light in the first biological window (at ca. 800 nm) due to the ^3^H_4_ → ^3^H_6_ optical transition.^18,26^ Furthermore, various nanomaterials doped with Eu^3+^, which exhibit red luminescence, have been reported for potential biomedical applications (optical imaging, PDT, various sensors, and so forth); however, the lack of scientific studies on the upconversion-driven luminescence of these ions indicates that such Eu^3+^-doped nanoparticles (NPs) typically require UV excitation, limiting their applicability in biosciences.^30−37^ It is important to note that introducing Eu^3+^ into the chemical composition of UCNPs could provide the advantage of their long luminescence decay times, typically in the order of milliseconds, facilitating UCNP detection during bioimaging by overcoming protein autofluorescence, which has decay times in the order of nanoseconds.^38^ Hence, by selecting a suitable chemical composition of UCNPs, it is possible to achieve multicolor luminescence with emission peaks of different natures, which enables the creation of multimodal UCNPs, allowing single-type particles to be utilized across a wide range of biorelated applications such as protein autofluorescence-free bioimaging, localized photodynamic therapy, targeted light-mediated chemotherapy, and more.

This article presents the synthesis and characterization of NaGdF_4_:Eu^3+^ (Eu^3+^ = 5, 10, 15, 20, 25, 30, and 50 mol %) core, NaGdF_4_:15%Eu^3+^@NaGdF_4_:49%Yb^3+^,1%Tm^3+^ core–shell, and NaGdF_4_:15%Eu^3+^@NaGdF_4_:49%Yb^3+^,1%Tm^3+^@NaGdF_4_:5%Yb^3+^,40%Nd^3+^ core–shell–shell nanoparticles that can be excited using both UV and NIR radiation. Their structural and morphological properties, including the crystal structure, particle shape, and size, are discussed in detail. Additionally, the spectroscopic properties of these nanomaterials, such as emission spectra and decay curves under excitation employing up to four different wavelengths (272, 394, 808, and 980 nm), were thoroughly analyzed. Finally, the colloidal stability of core–shell–shell nanoparticles in organic, aqueous, and biological media, along with the findings from biocompatibility studies, is evaluated and discussed.

## Experimental Section

### Materials

Gadolinium(III) acetate hydrate (99.9%, Alfa Aesar), neodymium(III) acetate hydrate (99.9%, Alfa Aesar), ytterbium(III) acetate hydrate (99.9%, Alfa Aesar), europium(III) acetate hydrate (99.9%, Alfa Aesar), and thulium(III) acetate hydrate (99.9%, Alfa Aesar) were dissolved in deionized water to obtain 0.2 M solutions. These solutions were filtered using 0.2 μm PES syringe filters (ROTH, Chromafil) before use. Oleic acid (OA, 90% technical grade, Alfa Aesar), 1-ocatadecene (ODE, 90% technical grade, Alfa Aesar), sodium hydroxide (NaOH, Eurochemicals), ammonium fluoride (NH_4_F, 99%, Eurochemicals), hydrochloric acid solution (HCl, 36.5%, Eurochemicals), methanol (MeOH, HPLC, Eurochemicals), *n*-hexane (Hex, HPLC, Eurochemicals), cyclohexane (cHex, HPLC, Eurochemicals), diethyl ether (Et_2_O, HPLC, Eurochemicals), and acetone (99.8%, Eurochemicals) were used as received, unless otherwise specified.

### Synthesis of NaGdF_4_:Eu^3+^ Core Nanoparticles

A series of NaGdF_4_:Eu^3+^ samples with different Eu^3+^ molar concentrations (Eu^3+^ = 5, 10, 15, 20, 25, 30, and 50 mol %) were synthesized according to the procedure described below.

Stoichiometric amounts of freshly prepared aqueous solutions (0.2 M) of gadolinium(III) acetate (Gd(OAc)_3_) and europium(III) acetate (Eu(OAc)_3_) were poured into a 50 mL three-necked round-bottomed flask and dried to a solid at 90–95 °C. The flask was then cooled to room temperature, and a mixture of the dry acetates was dispersed in methanol (3 mL) under vigorous stirring. Subsequently, 10 mL of OA and 15 mL of ODE were added to the dispersion of the acetates, and the flask was placed in a heating mantle equipped with a PID temperature controller and a glass-coated thermocouple. The reaction mixture was kept under an Ar atmosphere and gradually heated to 120 °C to remove methanol and any traces of moisture. When the temperature reached 120 °C, the vacuum line was connected, and the reaction solution was maintained at 120 °C under reduced pressure (15 mbar) for 15 min, followed by raising the temperature to 140 °C. The vacuum line was then disconnected, and the flask was filled with Ar. Next, the temperature was increased to 150 °C and maintained for 40 min under the Ar atmosphere. After this step, the flask was cooled down to room temperature, and the prepared solutions of NaOH (1 M in MeOH, 2.5 mL, 2.5 mmol) and NH_4_F (0.4 M in MeOH, 10 mL, 4 mmol) were mixed, shaken for 15 s, and poured into the reaction mixture at once. The obtained mixture was heated to 50 °C and maintained at this temperature for 30 min. Subsequently, the temperature was gradually increased to 120 °C to remove methanol from the reaction mixture. When the temperature reached 120 °C, the vacuum line was connected, and the reaction solution was maintained at 120 °C under reduced pressure (15 mbar) for 30 min. The temperature was then increased to 310 °C and maintained for 1 h (under an Ar atmosphere). Afterward, the reaction mixture was cooled to room temperature and poured into an excess of an acetone/hexane mixture (4:1 v/v, 150 mL). Nanoparticles were collected by centrifugation at 10,000 rpm for 10 min, followed by three other washing steps: acetone, acetone/DI water mixture (1:1 v/v), and acetone again. It should be noted that the particles were collected by centrifugation (10,000 rpm, 10 min) after every wash. Finally, the collected particles were redispersed in 20 mL of cyclohexane and used as a stock solution. The concentrations of the stock solutions were determined gravimetrically.

### Synthesis of Core–Shell and Core–Shell–Shell Nanoparticles

Core–shell and core–shell–shell nanoparticles with chemical compositions of NaGdF_4_:15%Eu^3+^@NaGdF_4_:49%Yb^3+^,1%Tm^3+^ and NaGdF_4_:15%Eu^3+^@NaGdF_4_:49%Yb^3+^,1%Tm^3+^@NaGdF_4_:5%Yb^3+^,40%Nd^3+^, respectively, were synthesized according to the procedure described below.

Depending on the chemical composition of the first or second shell, stoichiometric amounts of freshly prepared aqueous solutions of the required lanthanide acetates (gadolinium(III) acetate (Gd(OAc)_3_, 0.2 M), thulium(III) acetate (Tm(OAc)_3_, 0.05 M), ytterbium(III) acetate (Yb(OAc)_3_, 0.2 M), and neodymium(III) acetate (Nd(OAc)_3_, 0.2 M)) were poured into a 50 mL three-necked round-bottomed flask and dried to a solid at 90–95 °C. The following synthesis steps are identical to those described in the previous section (Synthesis of NaGdF4:Eu3+ Core Nanoparticles). As 40 min under 150 °C temperature passed, the reaction mixture was cooled down to room temperature, and 10 mL of the stock solution of obtained core particles (NaGdF_4_:15%Eu^3+^; in the case of synthesis of core–shell NPs) or 5 mL of the stock solution of core–shell particles (NaGdF_4_:15%Eu^3+^@NaGdF_4_:49%Yb^3+^,1%Tm^3+^; in the case of synthesis of core–shell–shell NPs) was added to the mixture. The cyclohexane was removed using reduced pressure (at 120 °C), and the reaction mixture was cooled down to room temperature once again before adding the prepared solutions of NaOH (1 M in methanol, 2.5 mL, 2.5 mmol) and NH_4_F (0.4 M in MeOH, 10 mL, 4 mmol). The remaining synthesis and purification procedures for obtaining the core–shell (NaGdF_4_:15%Eu^3+^@NaGdF_4_:49%Yb^3+^,1%Tm^3+^) and core–shell–shell (NaGdF_4_:15%Eu^3+^@NaGdF_4_:49%Yb^3+^,1%Tm^3+^@NaGdF_4_:5%Yb^3+^,40%Nd^3+^) nanoparticles were identical to those of the core nanoparticles (see previous section).

### Procedure for the Removal of Oleate Ligands from the Surface of Nanoparticles

Five mL portion of nanoparticle stock solution in cyclohexane was poured into a 50 mL centrifuge tube, mixed with a 5-fold amount of acetone (25 mL), and centrifuged at 10,000 rpm for 15 min. The collected particles were mixed with acidified deionized water (15 mL, pH 2.75, adjusted with HCl) and vigorously stirred for 4 h at room temperature. Subsequently, 10 mL of diethyl ether was added, and the aqueous/organic solution was mixed. The separated aqueous phase, containing the oleate-free NPs, was washed twice with diethyl ether to completely remove the residual oleic acid. The particles were then precipitated with acetone (1:3 v/v) and collected by centrifugation at 12,000 rpm for 30 min. Finally, the collected oleate-ligand-free nanoparticles were redispersed in 10 mL of deionized water and stored at 4 °C for further experiments. The concentrations of the aqueous NP dispersions were determined gravimetrically.

### X-ray Diffraction (XRD) Measurements

The crystal phase and purity of the prepared nanoparticles were examined by the XRD technique. XRD patterns were recorded using a Rigaku MiniFlexII (Rigaku, Japan) diffractometer operating in Bragg–Brentano geometry in a 5° ≤ 2θ ≤ 80° range under Ni-filtered Cu K_α_ radiation (scanning step width: 0.02°, scanning speed: 5°/min).

### Scanning Electron Microscopy (SEM)

The size and morphology of the synthesized particles were evaluated from SEM images taken with a field-emission scanning electron microscope Hitachi SU-70 (Hitachi, Japan) using an electron accelerating voltage of 5 kV. Particle size and size distribution were evaluated from SEM images using ImageJ v1.8.0 software and manually measuring the diameters of 50 random particles per sample.

### Measurements of Excitation and Emission Spectra

Excitation and emission spectra were recorded using an Edinburgh Instruments FLS980 spectrometer (Edinburgh Instruments, UK), equipped with double-grating Czerny–Turner excitation and emission monochromators, a 450 W Xe arc lamp, continuous-wave diode lasers (808 and 980 nm), and a single-photon counting photomultiplier (Hamamatsu R928P). When measuring excitation spectra, the λem was set to 613 nm, whereas excitation and emission slits were set to 1 and 5 nm, respectively. Emission spectra were recorded using 272 and 394 nm Xe lamp radiation (excitation and emission slits were set to 5 and 1 nm, respectively) as well as 808 and 980 nm laser radiation (for core–shell and core–shell–shell particles; emission slit was set to 0.5 nm, and nominal laser power was set to 1 W). Each spectrum was recorded with a 0.5 nm step width and a 0.2 s dwell (integration) time, with the sample (colloidal dispersion of nanoparticles in cyclohexane or DI water) under continuous stirring. The concentration of nanoparticles in each sample was 1 mg/mL. Emission spectra were corrected for instrument response using a correction file provided by Edinburgh Instruments. Excitation spectra were corrected with a reference detector.

### Measurements of Colloidal Stability in Different Aqueous Media

The colloidal stability of the NPs dispersed in different media was evaluated by measuring the change in the integral emission intensity as a function of time. The colloidal stability of oleate-free core–shell–shell NPs in three different media (slightly acidic (pH 5.5) and slightly basic (pH 7.4) aqueous (obtained by addition of HCl or NH_4_OH to DI water), and standard cell growth media DMEM supplemented with 10% fetal bovine serum (FBS), 100 U/ml penicillin, and 100 mg/mL streptomycin (pH 7.4)) was evaluated by analyzing their emission spectra as a function of time (0, 1, 2, 3, 4, 5, 6, 7, 8, and 24 h). Emission spectra were recorded using an Edinburgh Instruments FLS980 spectrometer under 808 nm laser radiation (the measurement details are identical to those described in the section “Measurements of Excitation and Emission Spectra”). The concentration of core–shell–shell NPs in each sample was 1 mg/mL. Throughout the measurements or between measurements, the samples were not disturbed or stirred.

### Measurements of Zeta Potential

The zeta potential of core–shell–shell nanoparticles in aqueous media (the concentration of NPs in each sample was 1 mg/mL) at different pH values (in the range from 4 to 9) was measured using a Zetasizer Nano ZS (Malvern, UK), equipped with a 4 mW He–Ne laser emitting at a wavelength of 632.8 nm. The zeta potential values were calculated from the electrophoretic mobility using the Smoluchowski model at 25 °C (the zeta potential distribution data were analyzed using the Zetasizer v.8.02 software from Malvern).

### Evaluation of Viability

The human breast cancer cell line MDA-MB-231 (purchased from the American Type Culture Collection) was used as an in vitro model for cellular biocompatibility experiments. The cells were cultured in a Dulbecco’s modified eagle medium (DMEM), supplemented with 10% (v/v) fetal bovine serum, 100 U/ml penicillin, and 100 μg/mL streptomycin (all from Corning, USA). The cells were maintained at 37 °C in a humidified atmosphere containing 5% CO_2_. The cells were routinely subcultured 2–3 times per week in 25 cm^2^ cells culture flasks.

For the lactate dehydrogenase (LDH) detection assay, MDA-MB-231 cells were seeded in a 96-well plate (TPP, Switzerland) at a density of 1.5 × 10^3^ cells/well. After 24 h, the old medium was replaced with fresh medium containing different concentrations of UCNPs (from 0.001 to 0.2 mg/mL), while medium alone without UCNPs was used as a control. The cells were then incubated for 24 h in the dark. The following day, the CyQUANT LDH Cytotoxicity Assay Kit (Thermo Scientific, USA) was used to detect the extracellular appearance of LDH. The concentration of extracellular LDH was quantified by measuring the absorbance at 490 and 630 nm using a plate-reading spectrophotometer (800TS microplate reader, BioTek, USA). After obtaining the absorbance values, they were recalculated as percentage values of cytotoxicity according to the protocol. For better data representation, the estimated cytotoxicity (%) was calculated as the viability of the cells (viability (%) = 100% – cytotoxicity (%)). Data are expressed as mean ± standard deviation (SD). The statistical significance of the differences between the studied groups was assessed using a two-tailed independent Student’s *t-*test at the 95% confidence level. Significance was set at *p* < 0.05.

## Results and Discussion

**Figure 1:**
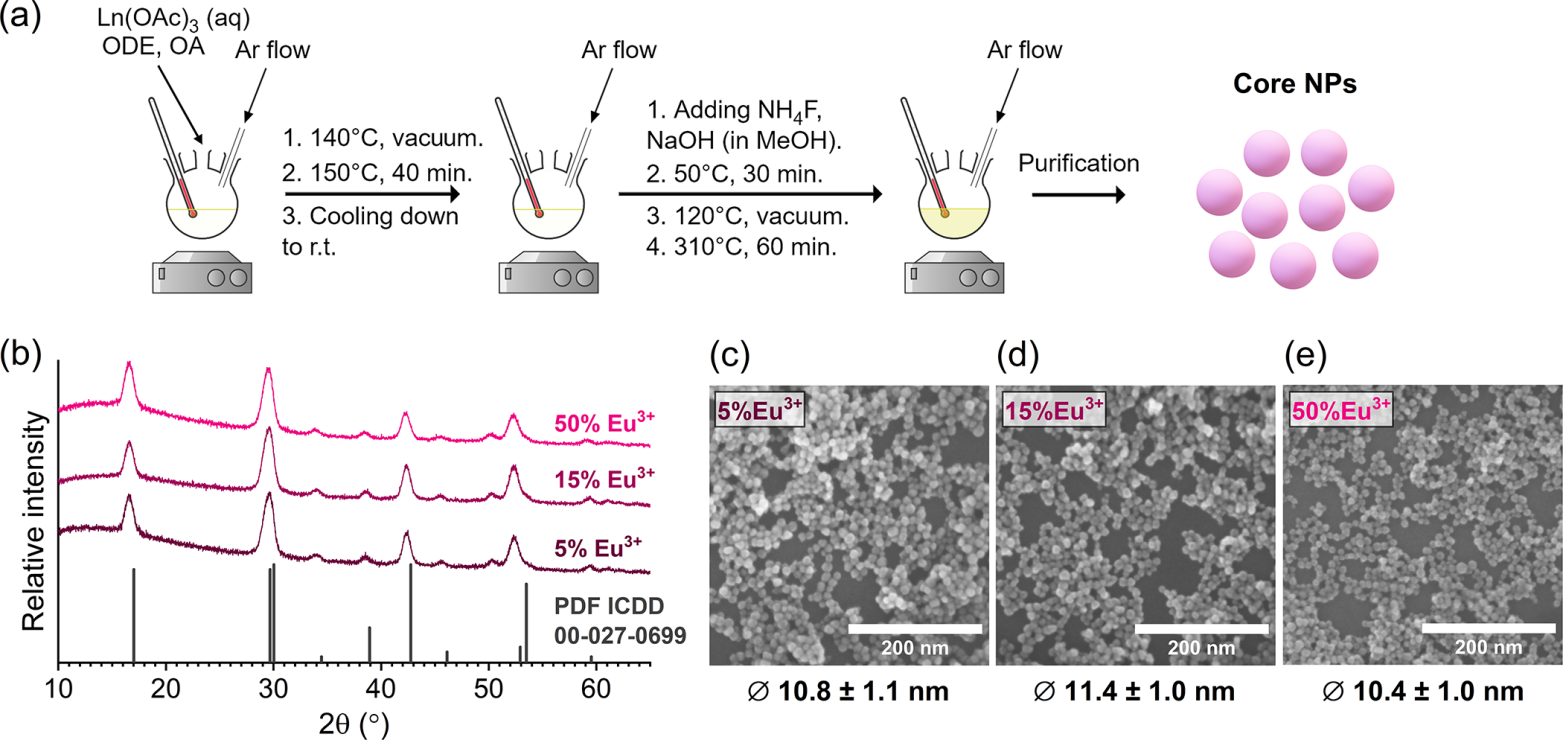
Synthesis scheme of NaGdF_4_:Eu^3+^ core NPs (a). XRD patterns of Eu^3+^-doped NaGdF_4_ NPs (Eu^3+^ = 5, 15, and 50 mol %) with the reference pattern of hexagonal NaGdF_4_ (PDF ICDD 00-027-0699) (b). SEM images of NaGdF_4_:5%Eu^3+^ (c), NaGdF_4_:15%Eu^3+^ (d), and NaGdF_4_:50%Eu^3+^ (e) NPs.

Thermal decomposition is the most popular synthesis method for obtaining lanthanide-doped NaGdF_4_ nanoparticles. This method involves precursors that decompose upon heating of the reaction mixture, leading to the formation of nanoparticles. It also allows simple control of nanoparticle size and usually yields a uniform size distribution.^38^ Lanthanide trifluoroacetate salts are usually used as precursors because they act as sources for both metal and fluoride ions; however, during the decomposition of trifluoroacetate ions, toxic fluorinated and oxy-fluorinated carbon species are generated.^39^ Therefore, a modified version of this synthesis route was chosen for this study to avoid harmful decomposition products. In brief, lanthanide acetates were transformed into lanthanide oleates by treatment with oleic acid at elevated temperatures. The obtained lanthanide oleates served as lanthanide precursors in the NP synthesis (the acetic acid formed during the anion exchange in the lanthanide salts was removed from the reaction mixture under reduced pressure).^40^ However, using this technique, the fluoride source required for the formation of NPs with the general formula NaGdF_4_:Ln^3+^ must be added separately (in the case of this study, NH_4_F was used as the fluoride source). Since the NP synthesis no longer involves the decomposition step of any precursor, this method cannot be called thermal decomposition. A more accurate name for this synthesis route is thermal coprecipitation, wherein the growth of NPs during synthesis at elevated temperatures (>300 °C) is based on Oswald ripening. Figure 1a shows the principal steps of Eu^3+^-doped NaGdF_4_ NP synthesis, which were used to produce a series of samples with different Eu^3+^ concentrations (NaGdF_4_:x%Eu^3+^; x = 5, 10, 15, 20, 25, 30, and 50 mol %). The structural properties of the NaGdF_4_:Eu^3+^ samples were investigated using XRD and SEM. The XRD patterns of all Eu^3+^-doped NaGdF_4_ NPs matched well with the reference pattern (PDF ICDD 00–027–0699), and no additional peaks were observed, indicating that all the prepared samples had a hexagonal crystal structure (space group P6̅ or P6_3/m_) with no impurities (see Figures 1b and S1).^41,42^ A slight shift of diffraction peaks of NaGdF_4_:Eu^3+^ NPs toward smaller angles (if compared with the reference pattern of NaGdF_4_) was observed, which indicates an increase in lattice parameters upon replacing some of the Gd^3+^ ions (r(Gd^3+^)^IX^ = 1.107 Å) with Eu^3+^ ions, which are, in fact, larger in size (r(Eu^3+^)^IX^ = 1.120 Å); therefore, such a shift is not surprising.^43,44^ Moreover, broadening of the diffraction peaks is clearly visible, which suggests a nanoscale particle size, which was confirmed by SEM images of the NaGdF_4_:Eu^3+^ samples (Figures 1c–e and S2).^45^ SEM images demonstrated that each sample of NPs consisted of unimodal spherical nanoparticles (cores) with an average size varying between 10.4 and 11.5 nm (the average size of each sample is provided in Table S1). Thus, XRD and SEM analyses of the Eu^3+^-doped NaGdF_4_ samples confirmed that each of them consists of monodisperse core nanoparticles with a hexagonal crystal structure regardless of the Eu^3+^ concentration.

**Figure 2:**
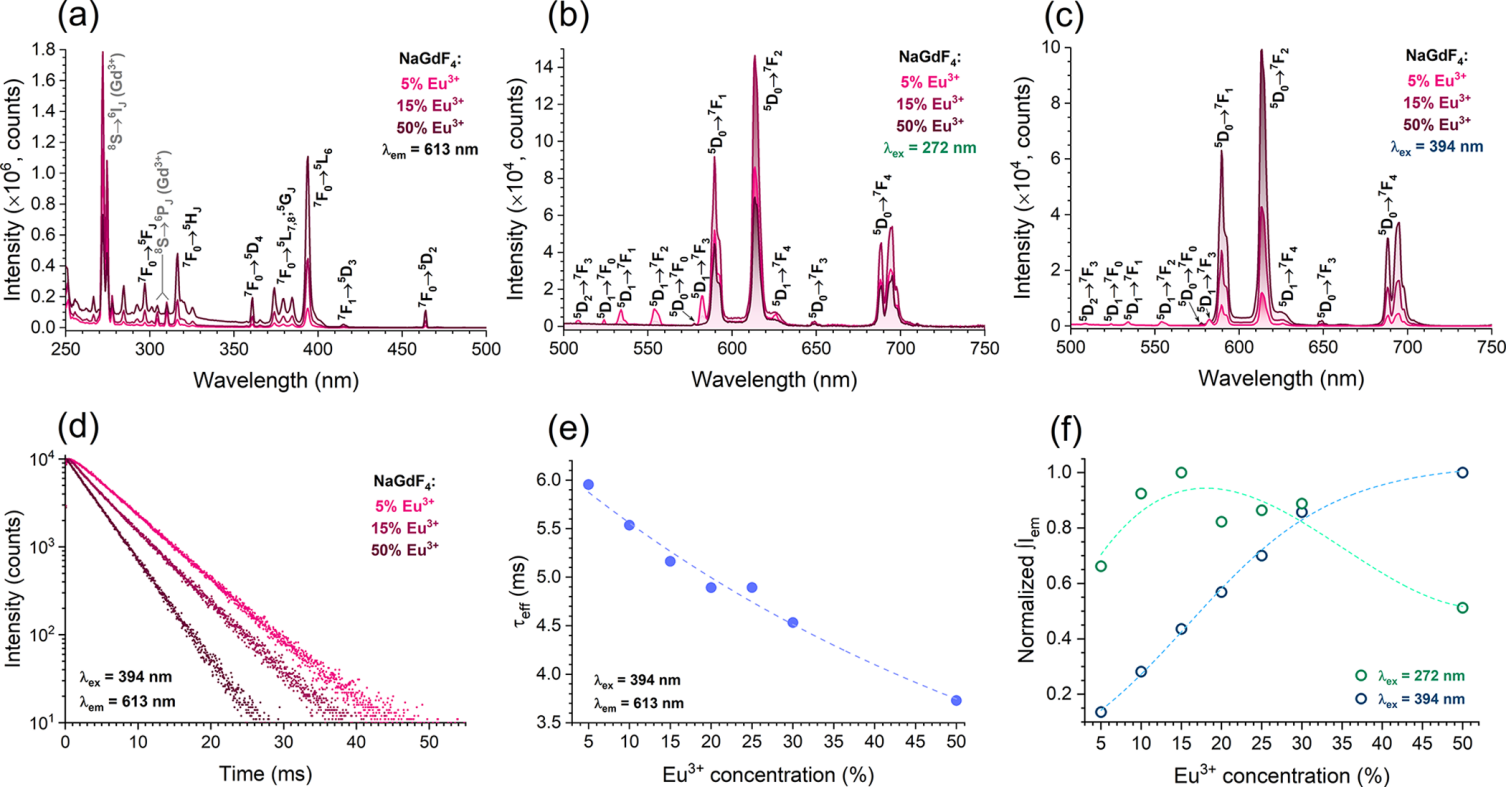
Excitation spectra (a), emission spectra under 272 (b) and 394 nm (c) excitation, and PL decay curves (d) of NaGdF_4_:Eu^3+^ NPs dispersed in cyclohexane. PL lifetime values (e) and normalized integrated emission intensity (f) of the synthesized NPs (dispersed in cyclohexane) as a function of the Eu^3+^ concentration.

The photoluminescence (PL) properties of the NaGdF_4_:Eu^3+^ NPs were investigated by analyzing their excitation and emission spectra as well as their PL decay curves. Figure 2a–d shows the optical characteristics of the three selected samples doped with 5, 15, and 50 mol % Eu^3+^. For the excitation and emission spectra or PL decay curves of samples doped with other concentrations of Eu^3+^, please refer to the Supporting Information (Figures S3–S6). The excitation spectra of the NaGdF_4_:Eu^3+^ NPs doped with 5, 15, and 50 mol % Eu^3+^ are shown in Figure 2a. Each excitation spectrum consists of typical Eu^3+^ excitation lines, the highest intensity of which belongs to the ^7^F_0_ → ^5^L_6_ optical transition (ca. 394 nm). In addition, the intensity of the excitation lines increases with an increasing Eu^3+^ concentration. Moreover, the excitation lines originating from the ^8^S ground state of Gd^3+^ are also clearly distinguished and peak at ca. 272 nm (^6^I_J_ terminal levels) as well as ca. 304.5 and 310 nm (^6^P_J_ terminal levels), with the NaGdF_4_:15%Eu^3+^ sample demonstrating the highest intensity of the Gd^3+^ excitation transitions. This observation indicates that the photoluminescence of these NPs can be induced either directly through Eu^3+^ or, alternatively, through Gd^3+^. Therefore, the investigation of Eu^3+^ luminescence in the matrix of NaGdF_4_ NPs involved recording emission spectra using not only direct excitation (λ_ex_ = 394 nm, Figure 2c) but also Gd^3+^ → Eu^3+^ energy transfer (λ_ex_ = 272 nm, Figure 2b). In both cases, Eu^3+^ emission in the yellow to red spectral region was dominant, with the highest intensity emission line observed at ca. 613 nm (^5^D_0_ → ^7^F_2_ transition). Furthermore, the NaGdF_4_:15%Eu^3+^ sample demonstrated the highest overall emission intensity when excited using 272 nm radiation, while both lower and higher Eu^3+^ concentrations yielded lower overall emission intensities (Figure 2f). However, when NPs were excited using 394 nm radiation, the overall emission intensities increased with the increasing amount of Eu^3+^ and reached the highest value for the sample doped with 50% Eu^3+^. Regardless of the excitation wavelength chosen, Eu^3+^ optical transitions from the ^5^D_1,2_ levels were also observed (mainly in the 509–590 nm interval), and their intensities increased with decreasing amount of Eu^3+^ in the NPs. This tendency is not surprising because higher amounts of Eu^3+^ tend to favor concentration quenching of the ^5^D_1,2_ levels to the ^5^D_0_ level.^46,47^

Regarding the PL decay of NaGdF_4_:Eu^3+^ NPs, monoexponential decay curves (λ_em_ = 613 nm, λ_ex_ = 394 nm) were recorded for each sample (Figures 2d and S6). These curves were used to calculate the PL lifetime values (τ_eff_) using the following equation:
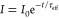
1where *I* stand for PL intensity at time *t*.^48^ As presented in Figure 2e and Table S2, the increase in Eu^3+^ concentration leads to a decrease in the PL lifetime values from ca. 5.95 ms (NaGdF_4_:5%Eu^3+^ sample) to ca. 3.73 ms (NaGdF_4_:50%Eu^3+^ sample), which was assigned to concentration quenching. A similar tendency was also observed in other inorganic hosts doped with Eu^3+^.^49−51^

**Figure 3:**
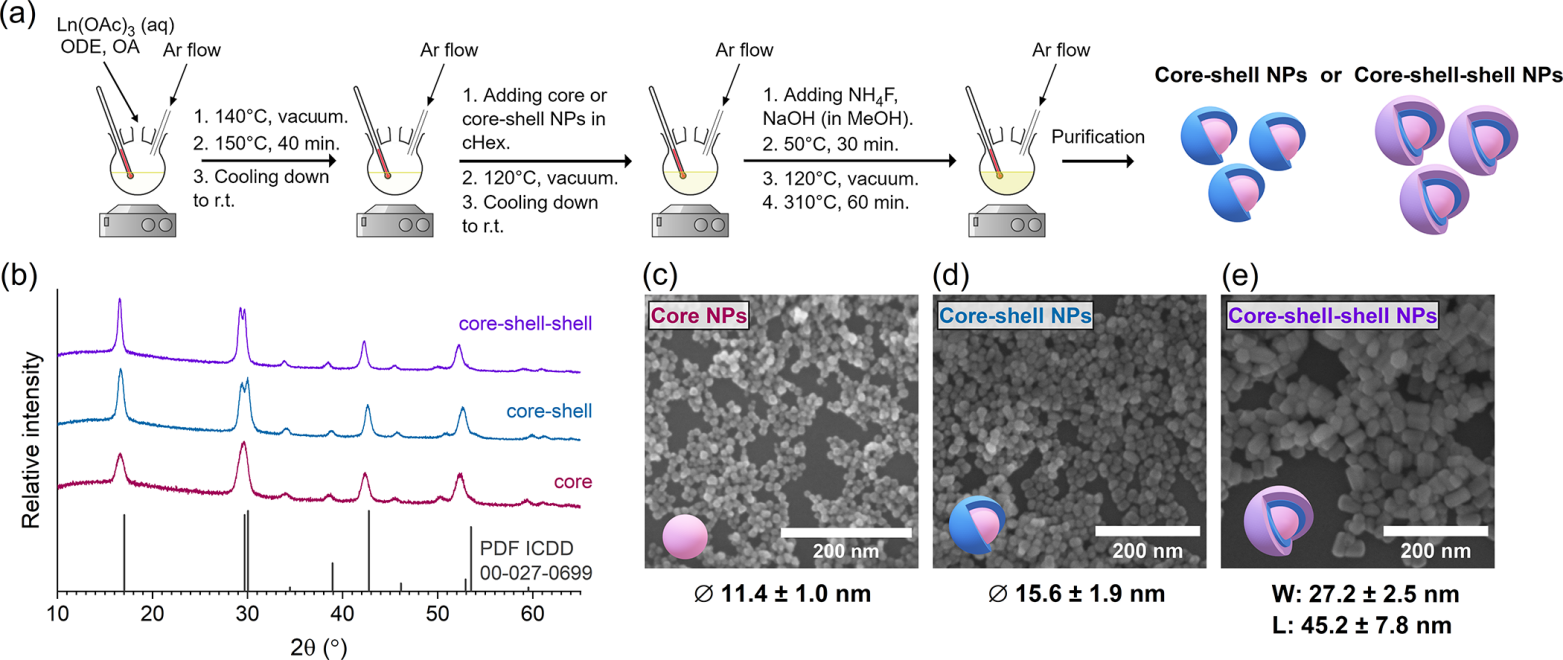
Synthesis scheme of core–shell or core–shell–shell NPs (a). XRD patterns of core, core–shell, and core–shell–shell NPs with a reference pattern of hexagonal NaGdF_4_ (PDF ICDD 00-027-0699) (b). SEM images of core (c), core–shell (d), and core–shell–shell (e) NPs.

As described above, NaGdF_4_:Eu^3+^ NPs can be excited with UV radiation. However, for these NPs to be applied in bioimaging, UV-based excitation is not appropriate because of its low permeability through tissues.^52^ Ideally, luminescent NPs dedicated to such purposes should be excited using NIR radiation that falls within the first or second biological window, which ensures excellent passage through the skin into target tissues.^53^ For NaGdF_4_:Eu^3+^ NPs to meet this requirement, their composition has been improved by introducing Yb^3+^, Tm^3+^, and Nd^3+^ into the system, leading to the development of NPs with more complex architectures, that is, core–shell or core–shell–shell. The upconversion emission of the samples was driven via complex energy transfer processes, which enabled the conversion of 980 and 808 nm radiation to UV–vis light through the Yb^3+^ → Tm^3+^ → Gd^3+^ → Eu^3+^ and Nd^3+^ → Yb^3+^ → Tm^3+^ → Gd^3+^ → Eu^3+^ energy transfer routes, respectively.^54^ Thus, NaGdF_4_:15%Eu^3+^ NPs were used as cores for the synthesis of NaGdF_4_:15%Eu^3+^@NaGdF_4_:49%Yb^3+^,1%Tm^3+^ core–shell and NaGdF_4_:15%Eu^3+^@NaGdF_4_:49%Yb^3+^,1%Tm^3+^@NaGdF_4_:5%Yb^3+^,40%Nd^3+^ core–shell–shell NPs (see the synthesis scheme in Figure 3a). NaGdF_4_:15%Eu^3+^ NPs were chosen for this study for they established the highest overall emission intensity when excited through Gd^3+^ ions, which are inevitably involved in energy transfer processes, regardless of whether 980 or 808 nm laser radiation was chosen to excite the NPs

XRD patterns of core, core–shell, and core–shell–shell NPs are provided in Figure 3b and reveal that all samples synthesized have a hexagonal crystal structure with no impurities present (reference—NaGdF_4_ XRD pattern PDF ICDD 00-027-0699). Moreover, with the addition of shells, the XRD signals became narrower and sharper, indicating an increase in particle size upon the addition of layers, which was confirmed by SEM analysis (Figure 3c–e). SEM images show that both the core and core–shell particles possess sphere-like shapes with average sizes ca. 11.4 and 15.6 nm, respectively. However, the core–shell–shell NPs appear to be cylinder-shaped, with an average width and length ca. 27.2 and 45.2 nm, respectively. Similar observations were made by other researchers and were explained as kinetically favored anisotropic shell growth, caused by certain facets being more reactive than others due to the lattice mismatches between the components of the inner and outer shells.^55−59^

**Figure 4:**
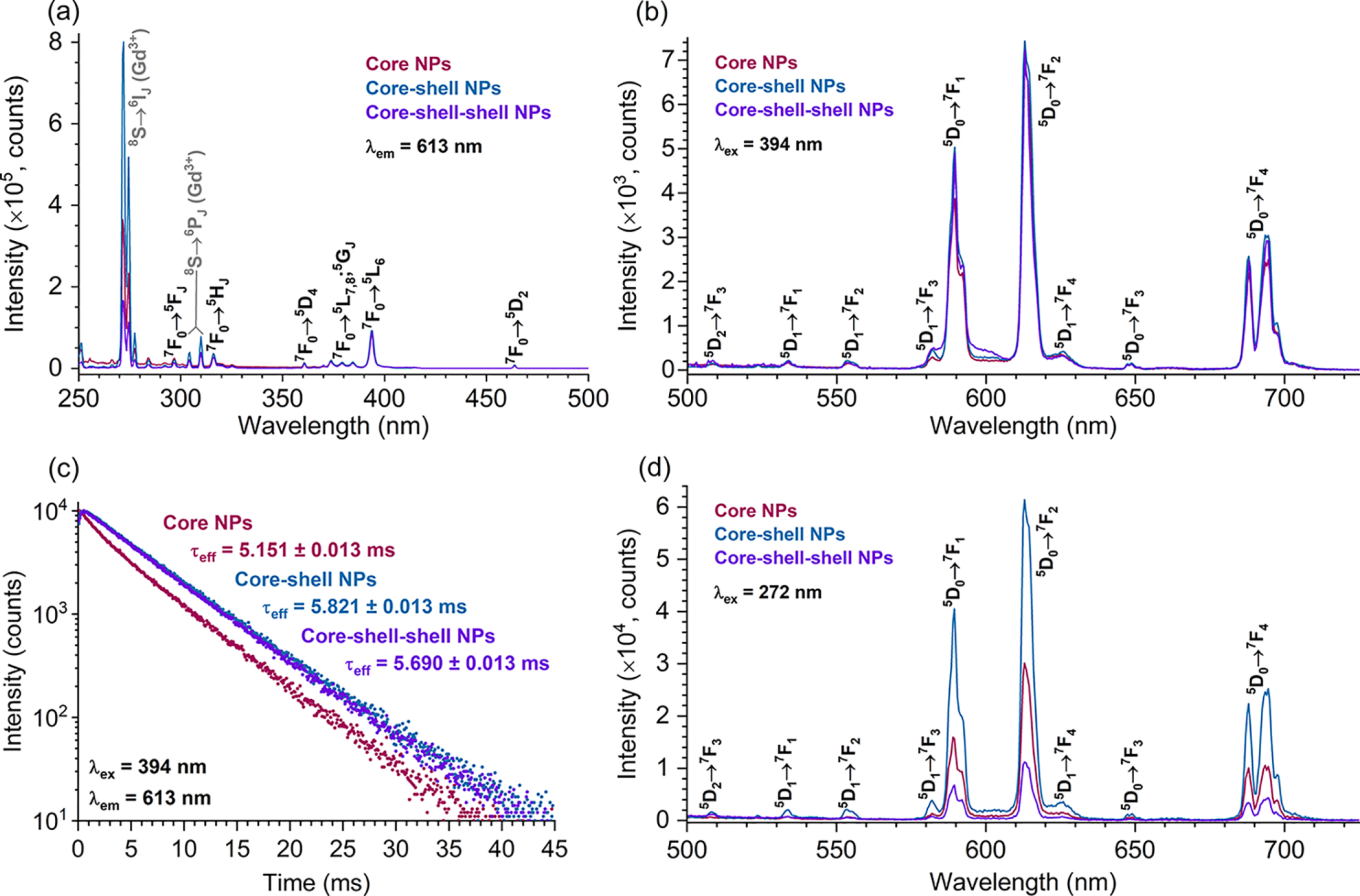
Excitation spectra (a) and emission spectra under 272 (b) and 394 nm (d) excitation, PL decay curves (c) of Eu^3+^ in core, core–shell, and core–shell–shell NPs dispersed in acidified DI H_2_O (adjusted with HCl, pH ca. 5.5). The concentration of Eu^3+^ in each sample was set to 20 mg/L.

After growing Ln^3+^-doped NaGdF_4_ shells onto NaGdF_4_:15%Eu^3+^ cores, the research proceeded with the evaluation of UV-enabled optical characteristics of the obtained core–shell and core–shell–shell NPs. For better comparison of excitation (λ_em_ = 613 nm) (Figure 4a) and emission spectra (λ_ex_ = 394 and 272 nm), the sample concentration for core, core–shell, and core–shell–shell particles was unified to a total Eu^3+^ concentration of 20 mg/L (according to ICP-OES data). The excitation spectra (λ_em_ = 613 nm) of the core, core–shell, and core–shell–shell NPs consist of characteristic Eu^3+^ and Gd^3+^ excitation lines, which have already been discussed. The emission spectra under UV excitation (λ_ex_ = 394 and 272 nm) of the shell-covered NPs are given in Figure 4b,d and show the optical transitions of Eu^3+^, which are analogous to those observed when investigating the NaGdF_4_:Eu^3+^ core samples. However, the intensities of the different samples varied with regard to the wavelength of the excitation source. The direct excitation to Eu^3+^ (λ_ex_ = 394 nm) resulted in nearly identical emission intensities between the samples. A slightly lower intensity for core samples was observed, which can be attributed to quenching effects caused by the surface Eu^3+^ ions interacting with the surrounding environment. This hypothesis is further supported by the slightly shorter calculated emission lifetimes (Figure 4c). In contrast, excitation through Gd^3+^ ions (λ_ex_ = 272 nm) led to far more intriguing results. Upon introducing the first shell around the core, a significant enhancement in emission intensity was observed. This improvement can be attributed to the shielding of Eu^3+^ ions from the environmental quenching processes. Notably, the thickness of the first outer shell (∼2 nm) does not interfere with the efficiency of energy transfer (ET) from Gd^3+^ to Eu^3+^. On the other hand, adding the second shell caused a significant increase in particle size (more than two-fold). As a result, the energy transfer from Gd^3+^ ions located in the outermost shell to Eu^3+^ ions becomes less efficient due to the increased amount of Gd–Gd energy migration cycles. During these cycles, Gd^3+^ → Gd^3+^ energy transfer and energy scattering, including back energy transfer processes, occur, leading to reduced excitation efficiency. This phenomenon is evident in the excitation spectra, where the excitation bands associated with Gd^3+^ for core–shell–shell particles show a significant decrease in intensity compared to core–shell counterparts (Figure 4a).

It is important to note that these structural changes and energy transfer pathways had a negligible effect on the lifetimes of the Eu^3+^ emission. The calculated values were virtually the same regardless of the applied excitation wavelength (Figures 4c and S7). Based on the results of these experiments, the following conclusions could be drawn: if Eu^3+^ ions are excited directly (λ_ex_ = 394 nm), the emission intensity is primarily dependent on the concentration of Eu^3+^. However, if excitation goes through Gd^3+^ (λ_ex_ = 272 nm), additional factors, including various Gd^3+^↔Gd^3+^ energy migration processes, must be considered, particularly in core–shell–shell structures, where the energy transfer pathway becomes more complex and less efficient.

**Figure 5:**
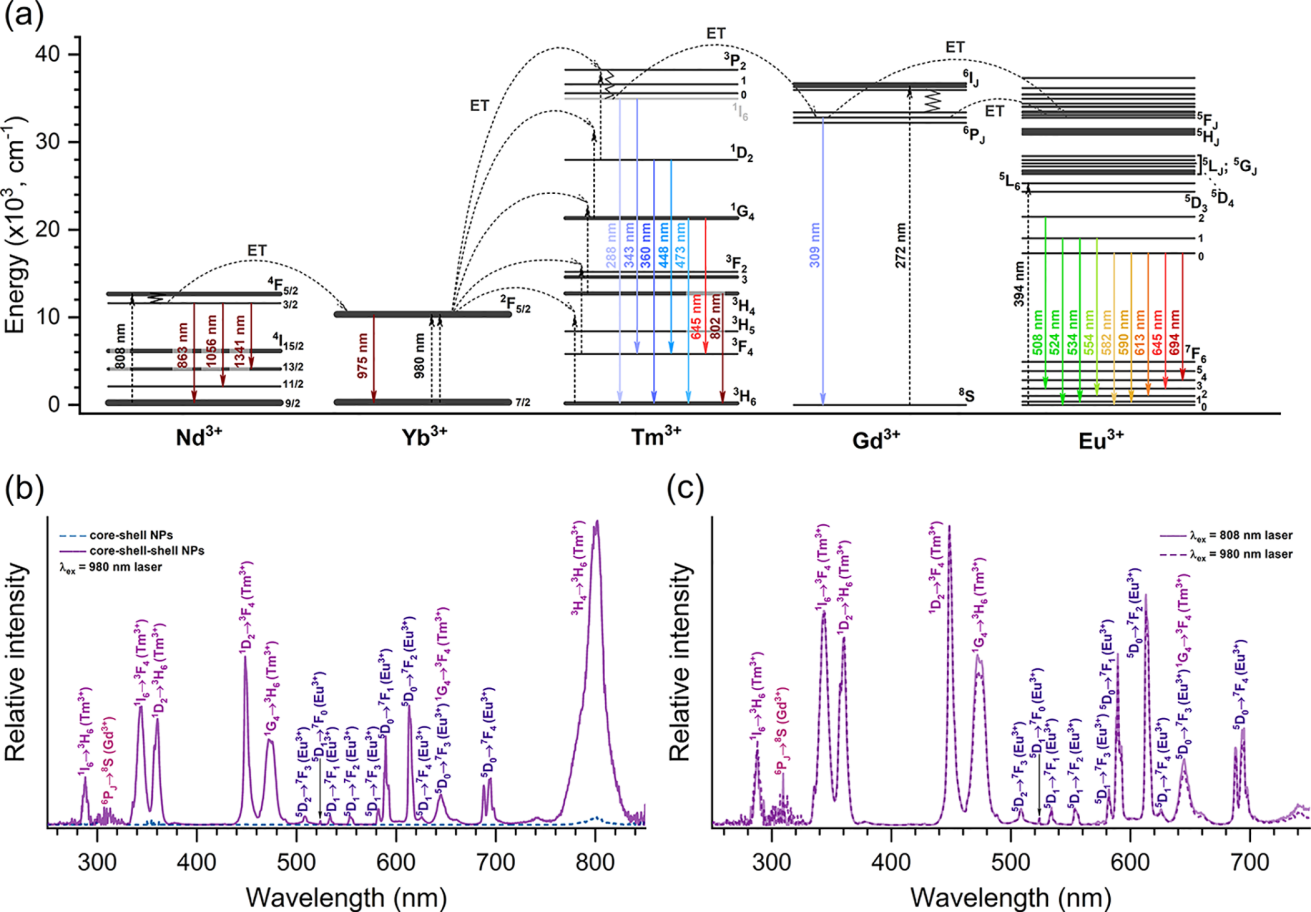
Energy levels and possible energy transfer (ET) routes between Nd^3+^, Yb^3+^, Tm^3+^, Gd^3+^, and Eu^3+^ ions (a). Emission spectra of core–shell and core–shell–shell NPs dispersed in cyclohexane under 980 nm laser radiation (b). Emission spectra of core–shell–shell NPs dispersed in cyclohexane under 808 and 980 nm laser radiation (c).

The presence of Yb^3+^ and Tm^3+^ ions within the structure of core–shell NPs introduces a wider array of photoluminescence characteristics compared to those of core NPs alone. Due to the compatible structure of energy levels, Yb^3+^ and Tm^3+^ are known as efficient upconversion pair that converts NIR radiation to blue or even NUV photons.^60^ As presented in Figure 5a, Yb^3+^ can absorb 980 nm photons and transfer them to adjacent Tm^3+^ ions, which emit the photons in the form of light ranging from UV to NIR. Alternatively, energy from higher levels of Tm^3+^ (^3^P_0,1,2_, ^1^I_6_) can be transferred to nearby Gd^3+^ ions, which can emit this energy as UV radiation or transfer it to Eu^3+^ ions, leading to their distinct emission lines in the green to red range of the visible spectrum. All of the energy transfer outcomes described above were practically established once the emission spectra (λ_ex_ = 980 nm laser) of the core–shell and core–shell–shell UCNPs were recorded (Figure 5b). Clearly, the overall emission intensity of the core–shell–shell NPs under 980 nm radiation was significantly higher compared to the emission from core–shell NPs. There are two possible explanations for this observation. First, the outer shell of core–shell–shell NPs reduces surface defects and protects optically active ions located in the inner shell and core from quenching caused by the surrounding media.^61^ Second, the outer shell of core–shell–shell NPs possesses sensitizer ions (Yb^3+^); therefore, the energy obtained from 980 nm laser radiation can easily migrate between Yb^3+^ ions contained in the outer and inner shells, resulting in efficient energy transfer to activator ions (Tm^3+^). Furthermore, the energy transfer from Yb^3+^ to Tm^3+^ was successfully confirmed, as evidenced by distinct optical transitions of Tm^3+^ ions. These include UV emissions from the ^1^I_6_ → ^3^H_6_, ^1^I_6_ → ^3^F_4_, and ^1^D_2_ → ^3^H_6_ transitions (with peaks at 287, 343, and 360 nm, respectively); blue emissions from the ^1^D_2_ → ^3^F_4_ and ^1^G_4_ → ^3^H_6_ transitions (peaking at 449 and 472 nm, respectively); red emission from the ^1^G_4_ → ^3^F_4_ transition (peaking at 645 nm, merging with the ^5^D_0_ → ^7^F_3_ transition of Eu^3+^); and NIR emission from the ^3^H_4_ → ^3^H_6_ transition (peaking at ca. 802 nm, with the highest intensity across the spectrum). Because of the Tm^3+^ → Gd^3+^ energy transfer, the emission lines originating from the Gd^3+^^8^S → 6P_J_ optical transition in the 300–320 nm range were also observed in the emission spectra. In addition, characteristic emission lines of Eu^3+^, which were observed in the emission spectra of core NPs and analyzed previously, were also detected in the emission spectra of core–shell and core–shell–shell NPs, with the emission line of the highest intensity peaking at ca. 613 nm (^5^D_0_ → ^7^F_2_ transition). Furthermore, for biomedical applications, excitation of UCNPs with 808 nm laser radiation is more desirable compared to that with 980 nm. This is due to the ability of 808 nm laser radiation to pass through cells or tissues more efficiently if compared to 980 nm radiation, leading to more detailed and accurate bioimaging possibilities.^62^ Therefore, the design of core–shell–shell NPs includes Nd^3+^ ions, which can absorb 808 nm laser radiation and transfer it to nearby Yb^3+^ ions with further energy transfer processes, as presented in Figure 5a and described previously. Figure 5c demonstrates the emission spectra of the core–shell–shell NPs under 980 and 808 nm laser radiation. The spectra are virtually identical in terms of overall intensity, confirming efficient Nd^3+^ → Yb^3+^ energy transfer.

**Figure 6:**
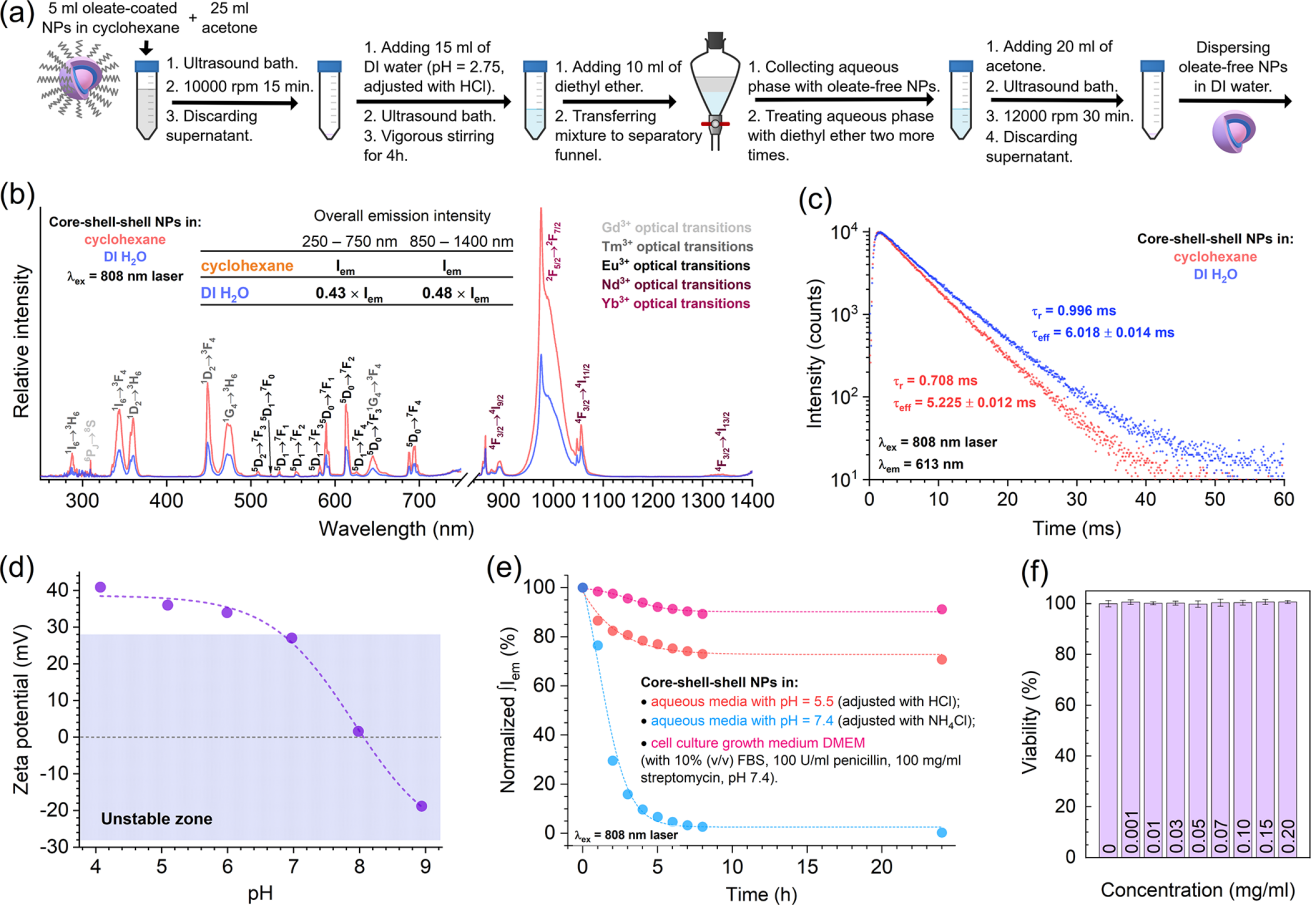
Procedure scheme for the removal of oleate ligands from the surfaces of NPs (a). Emission spectra of core–shell–shell NPs in cyclohexane and DI H_2_O under 808 nm laser radiation (b). PL decay curves of core–shell–shell NPs in cyclohexane and DI H_2_O (c). Zeta potential values of core–shell–shell NPs as a function of the pH of the aqueous media (d). Normalized integrated emission intensity of core–shell–shell NPs in various media as a function of time (e). Viability (determined via the LDH test) of MDA-MB-231 cells exposed to different concentrations of core–shell–shell NPs (f).

Despite the excellent optical properties of UCNPs, their direct application in biomedicine is limited due to the hydrophobic nature of their surface. After synthesis and purification, the nanoparticle surface was capped with oleate ligands. Oleic protecting ligands are beneficial during the synthesis of UCNPs, preventing their agglomeration, even at elevated temperatures. However, these ligands are responsible for NP agglomeration in aqueous environments, making the NPs inapplicable in most biorelated fields. To solve this issue, detachment of surface-bound OA was performed. The process involved acidic treatment of the produced NPs (as was established in a previous study and is illustrated in Figure 6a), ensuring NP dispersibility and stability in aqueous media.^19^Figure 6b shows the emission spectra of the oleate-capped core–shell–shell and bare UCNPs in cyclohexane and acidified DI H_2_O (pH 5.5, adjusted with HCl), respectively (λ_ex_ = 808 nm). Each emission spectrum, in the range of 250 to 750 nm, consists of the already indicated Gd^3+^, Tm^3+^, and Eu^3+^ optical transitions, covering most of the visible spectrum. Moreover, the emission of core–shell–shell NPs in the NIR region (850–1400 nm) was also recorded, revealing the optical transitions of Nd^3+^ and Yb^3+^ and indicating that not all the energy absorbed upon excitation participates in the upconversion process. The emission line with the highest intensity in the NIR region belongs to Yb^3+^ and peaks at ca. 975 nm (^2^F_5/2_ → ^2^F_7/2_ transition). The emission lines in the NIR region are of high significance because they ensure the possibility of detecting NPs in vivo via a noninvasive manner using an NIR camera.^63,64^ It is important to note that after the removal of OA ligands, the overall emission intensity of core–shell–shell NPs in aqueous solutions was reduced by approximately half compared to that of OA-capped NPs. However, the observed emission quenching is not surprising because water molecules located near the surface of such NPs are known to act as surface oscillators, significantly decreasing the emission of lanthanide ions.^65,66^

The PL decay curves (λ_ex_ = 808 nm laser, λ_em_ = 613 nm) of core–shell–shell NPs recorded in both cyclohexane and acidified DI H_2_O (pH 5.5, adjusted with HCl) were monoexponential (Figure 6c). The calculated PL rise time (τ_r_) and PL lifetime (τ_eff_) values in cyclohexane were τ_r_ = 0.708 ms and τ_eff_ = 5.225 ms, while in an aqueous environment, both values were larger: τ_r_ = 0.996 ms and τ_eff_ = 6.018 ms.

To evaluate the colloidal stability of the synthesized nanoparticles, the zeta potential was measured as a function of the pH of the aqueous media. Measuring the zeta potential (ζ) of bare core–shell–shell NPs (with OA chains removed) allowed us to predict their stability under various pH values in aqueous media (Figure 6d). According to DLVO theory, particles with |ζ| > 28 mV exhibit sufficient electrostatic repulsion to ensure their colloidal stability; however, these values are not absolute and depend on the pH and ionic strength of the media.^67^ The zeta potential value of the NPs was >28 mV at pH < 7, indicating that these NPs tend to agglomerate in neutral and slightly basic media. The determined isoelectric point (IEP), where the agglomeration of NPs occurs most rapidly, was pH 8. Furthermore, it is well-known that upon agglomeration of NPs, their emission intensity decreases; therefore, the colloidal stability of these NPs was evaluated through investigating the change in NP emission intensity over time in three different media: aqueous media with pH 5.5, pH 7.4, and cell culture growth media DMEM supplemented with 10% (v/v) of FBS (DMEM + FBS, 100 U/ml penicillin, 100 mg/mL streptomycin, pH 7.4). The lowest colloidal stability of the NPs was observed in aqueous media at pH 7.4. In this case, after 1 h, the emission intensity dropped to ca. 77%; after 4 h—to ca. 9.7%; after 8 h—to ca. 2.7%, and after 24 h—to ca. 0.3% compared to their initial intensity, indicating that almost all NPs were agglomerated. On the other hand, the UCNPs dispersed in slightly acidic aqueous medium (pH 5.5) showed good colloidal stability and maintained ca. 71% of their initial emission intensity even after 24 h of the experiment. The obtained colloidal stability results are in good agreement with the zeta potential measurements. UCNPs dispersed in slightly acidic aqueous media (pH 5.5) exhibited stronger electrostatic repulsion due to a more expressed surface charge (zeta potential value of ca. 37 mV) compared to those dispersed in neutral medium (pH 7.4, ca. 17 mV). Moreover, even though the pH value of the DMEM+FBS media was 7.4, the NPs contained within such an environment showed superior emission stability, that is, the emission intensity only dropped to ca. 91% when compared to the initial emission intensity of the sample. This is due to the proteins in DMEM media adsorbing onto the surface of the core–shell–shell NPs, forming a protein corona and providing additional steric hindrance, preventing nanoparticles from agglomeration. This results in better colloidal stability, and the emission intensity remains almost constant over time.^26,68^ Additionally, the formation of a protein corona on NPs in biological fluids plays an important role in their cellular uptake, affecting the possibilities of imaging, targeted drug delivery, and localized therapy.^69−71^

The biocompatibility of core–shell–shell NPs was investigated by evaluating the viability of MDA-MB-231 cells exposed to different concentrations of NPs (0 – control, 0.001, 0.01, 0.03, 0.05, 0.07, 0.10, 0.15, and 0.20 mg/mL; in DMEM supplemented with 10% (v/v) FBS). Viability values were obtained from data generated via two colorimetric cytotoxicity assays: LDH (which assesses the degree of plasma membrane damage done to cells by a material) and XTT (which detects the metabolic activity of cells undamaged by the material). Figure 6f shows the results obtained from the LDH assay, which revealed that there was no significant influence on the viability of MDA-MB-231 cells, regardless of the concentration of core–shell–shell NPs. The results obtained using the XTT assay (Figure S8) also demonstrated that MDA-MB-231 cells were statistically viable, regardless of the NPs concentration. Similar viability results obtained via two independent assays assured the biocompatibility of core–shell–shell NPs, which is high enough for further, more complex biological testing, leading to practical implementation of NPs in biorelated fields.

## Conclusions

In summary, this study describes the successful engineering of nanoparticles with a complex core–shell–shell architecture, exhibiting diverse luminescence lines across the NUV–vis-NIR spectral range (275–1350 nm). These nanoparticles possess a pure crystal structure and display a uniform size distribution. The removal of the protecting ligand from the nanoparticle surface facilitated their dispersibility in aqueous solutions, resulting in superior colloidal stability in cell growth media (DMEM+FBS). Furthermore, the nanoparticles demonstrated negligible toxicity, as cell viability remained unaffected, even at relatively high concentrations (200 μg/mL). Notably, the unusual upconversion luminescence of Eu^3+^ was achieved through complex energy transfer and migration processes within the Nd^3+^–Yb^3+^–Tm^3+^–Gd^3+^–Eu^3+^ ion system. The core–shell–shell nanoparticles could be excited by using four different wavelengths (272, 394, 808, and 980 nm), with Eu^3+^ upconversion emission offering multiple benefits. First, the long decay time helps avoid unwanted autofluorescence from biological tissues during UV excitation. Second, the inclusion of additional optical transitions in the red range of the visible spectrum enhances their suitability for bioimaging applications. Finally, the extended emission spectrum overlaps with clinically relevant photosensitizers, improving the generation of singlet oxygen (^1^O^·^) and ROS. Thus, these novel nanoparticles are promising candidates for use as effective photodynamic therapy nanoplatforms in cancer theranostics.

## Supporting Information Available

XRD patterns, SEM images, average width, excitation and emission spectra, PL decay curves, and PL lifetime values of NaGdF_4_:Eu^3+^ nanoparticles (PDF)

## Abbreviations

DLVO: Derjaguin–Landau–Verwey–Overbeek theory
DMEM: Dulbecco’s modified eagle medium
ET: energy transfer
FBS: fetal bovine serum
HPLC: high-performance liquid chromatography
IEP: isoelectric point
LDH: lactate dehydrogenase
MRI: magnetic resonance imaging
NIR: near-infrared
NPs: nanoparticles
NUV: near-ultraviolet
OA: oleic acid
ODE: 1-octadecene
PDT: photodynamic therapy
PID: proportional–integral–derivative controller
PL: photoluminescence
ROS: reactive oxygen species
SEM: scanning electron microscopy
UCNPs: upconverting nanoparticles
UV: ultraviolet
VIS: visible
XRD: X-ray diffraction
XTT: sodium 3′-[1-(phenylaminocarbonyl)-3,4-tetrazolium]-bis (4-methoxy6-nitro) benzenesulfonic acid hydrate
