# Supplementary material for: Development of Biocompatible, UV and NIR Excitable Nanoparticles with Multiwavelength Emission and Enhanced Colloidal Stability
Source: ACS Mater Au. 2025 Jan 1;5(2):353–64. doi: 10.1021/acsmaterialsau.4c00151 (PMC11907297; doi:10.1021/acsmaterialsau.4c00151)
Supplement: Supplementary file 1 — mg4c00151_si_001.pdf [file mg4c00151_si_001.pdf]

Supporting information

Development of Biocompatible, UV and NIR Excitable Nanoparticles with Multiwavelength Emission and Enhanced Colloidal Stability

Egle Ezerskyte[1,2], Greta Butkiene[2], Arturas Katelnikovas[1] and Vaidas Klimkevicius*[,1,2]

[1]Institute of Chemistry, Faculty of Chemistry and Geosciences, Vilnius University, Naugarduko 24, LT-03225 Vilnius, Lithuania

[2]Biomedical Physics Laboratory, National Cancer Institute, Baublio 3b, LT-08406 Vilnius, Lithuania

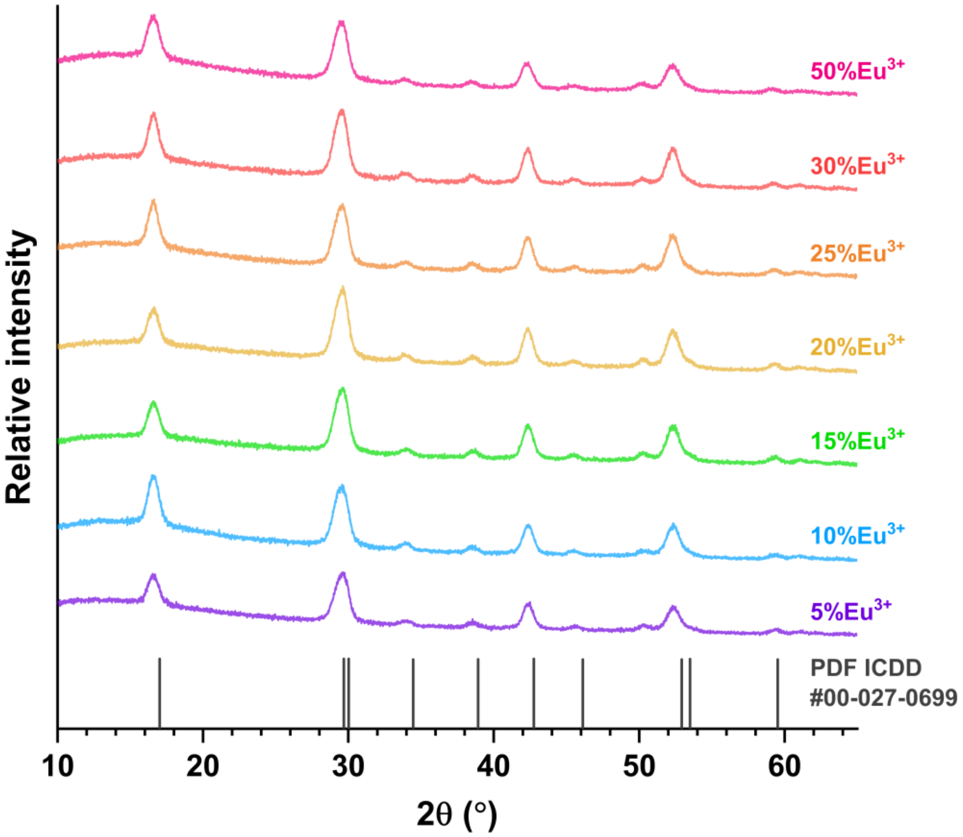

Figure S1. XRD patterns of $NaGdF_4:Eu^{3+}$ core NPs with reference pattern of hexagonal $NaGdF_4$ (PDF ICDD 00-027-0699).

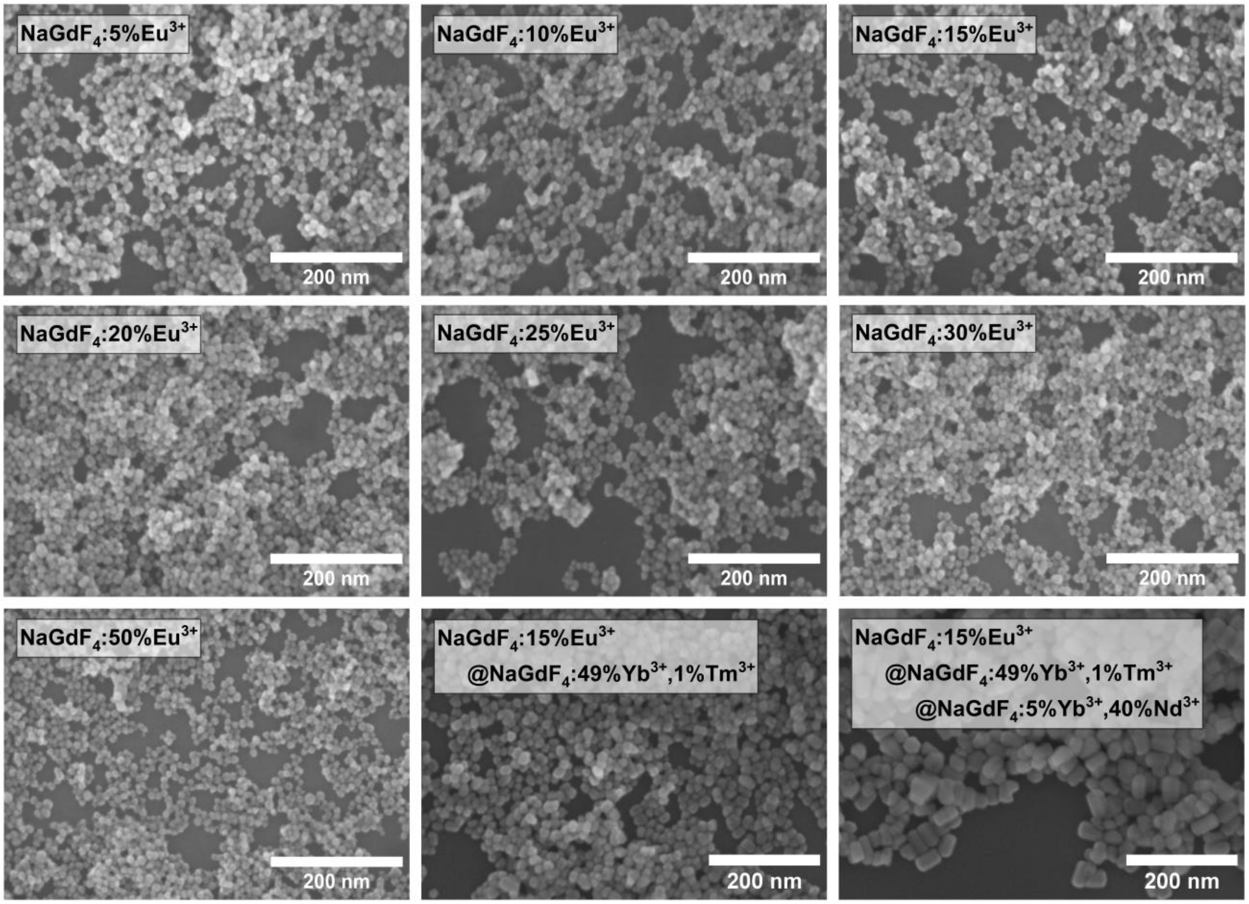

Figure S2. SEM images of core, core-shell and core-shell-shell samples synthesized within the framework of this study.

Table S1. Average width of $NaGdF_4:Eu^{3+}$ core NPs.

Sample	Width, nm	Standard deviation, nm
$NaGdF_4$:5%Eu^{3+}	10.8	1.1
$NaGdF_4$:10%Eu^{3+}	11.5	1.4
$NaGdF_4$:15%Eu^{3+}	11.4	1.0
$NaGdF_4$:20%Eu^{3+}	11.4	1.0
$NaGdF_4$:25%Eu^{3+}	10.9	1.2
$NaGdF_4$:30%Eu^{3+}	11.4	1.3
$NaGdF_4$:50%Eu^{3+}	10.4	1.0

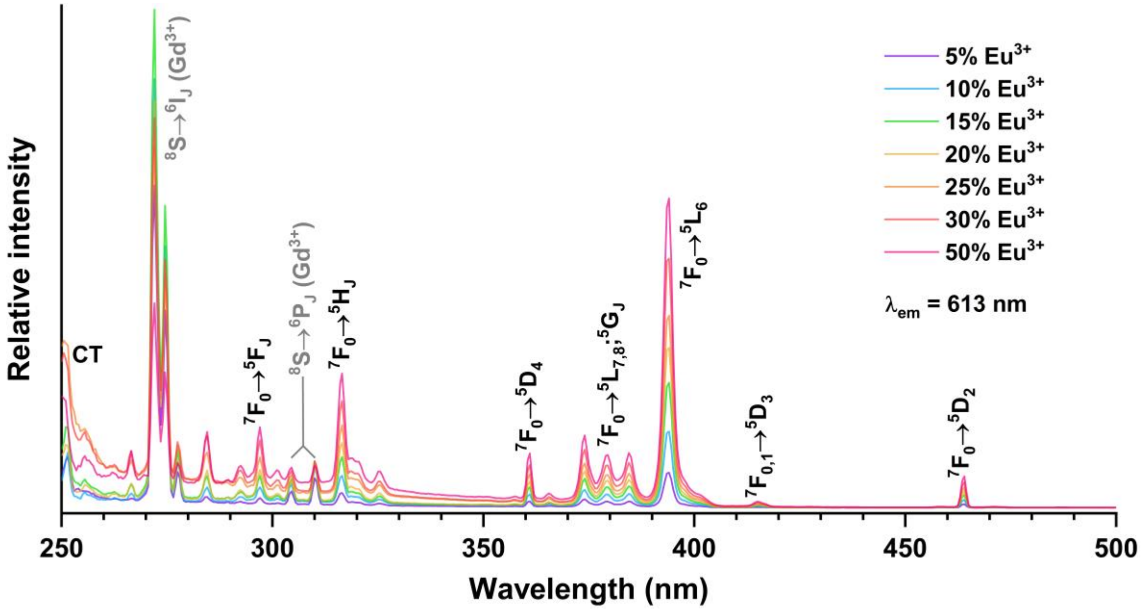

Figure S3. Excitation spectra of $NaGdF_4:Eu^{3+}$ core NPs (λ_{em} = 613 nm).

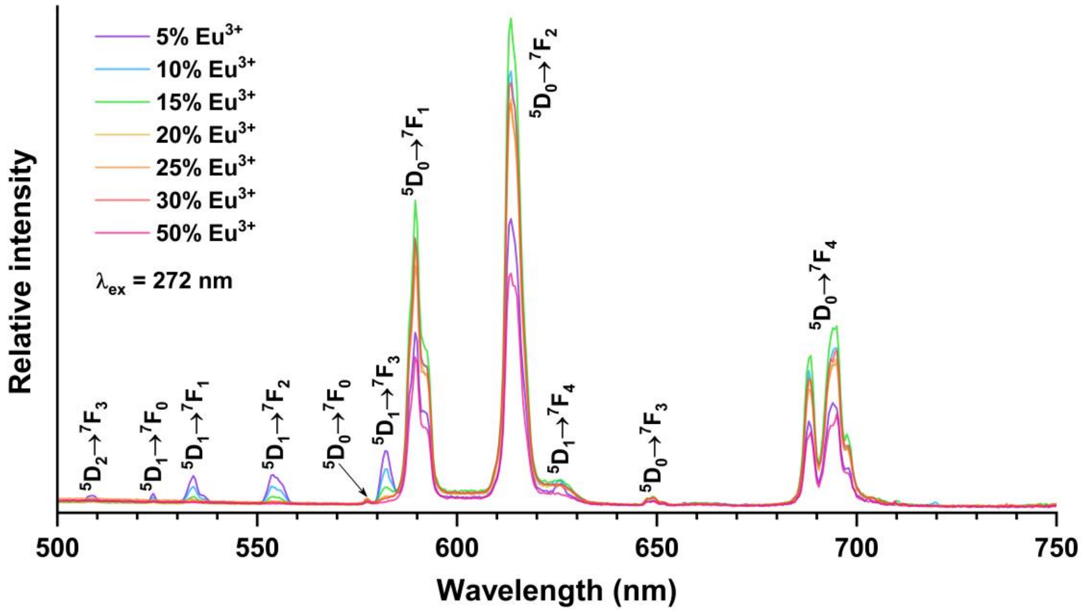

Figure S4. Emission spectra of $NaGdF_4:Eu^{3+}$ core NPs (λ_{ex} = 272 nm).

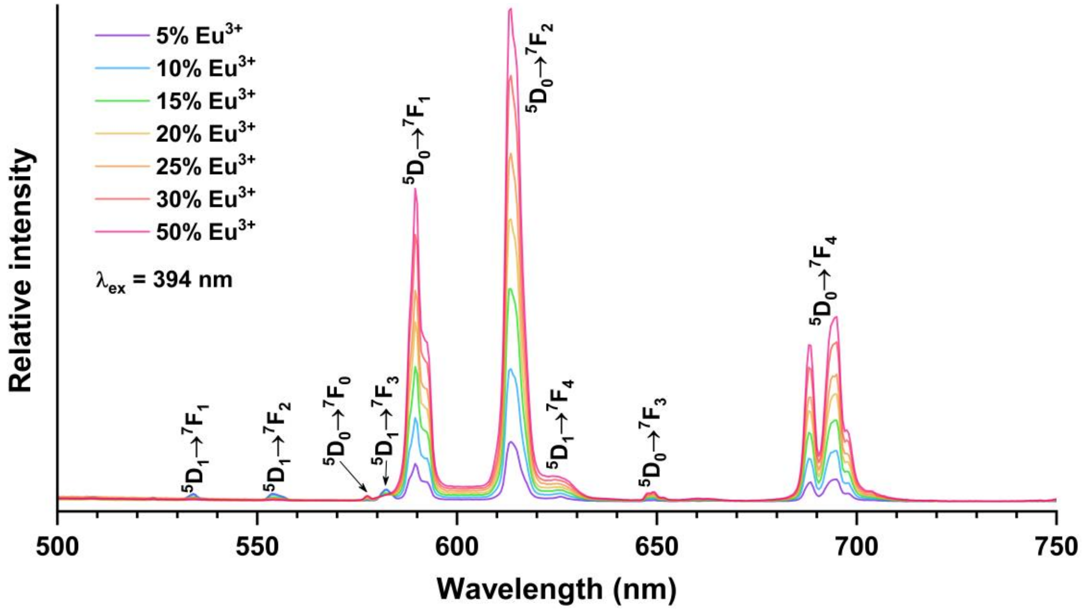

Figure S5. Emission spectra of $NaGdF_4$:Eu^{3+} core NPs (λ_{ex} = 394 nm).

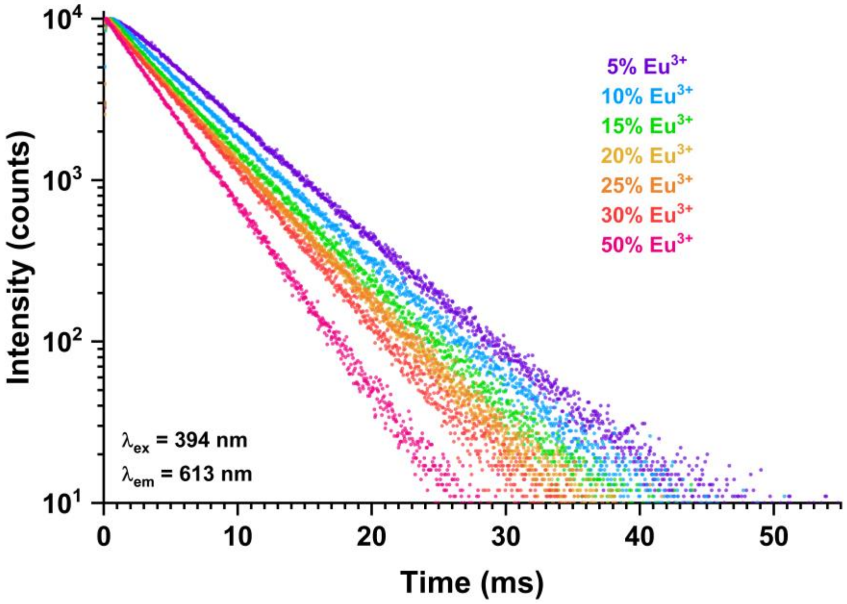

Figure S6. PL decay curves of $NaGdF_4$:Eu^{3+} core NPs (λ_{ex} = 394 nm, λ_{em} = 613 nm).

Table S2. PL lifetime values of $NaGdF_4:Eu^{3+}$ core NPs (λ_{ex} = 394 nm, λ_{em} = 613 nm).

**Sample**	**τ_{eff}, ms**	**Standard deviation, ms**
$NaGdF_4$:5%Eu^{3+}	5.954	0.006
$NaGdF_4$:10%Eu^{3+}	5.536	0.006
$NaGdF_4$:15%Eu^{3+}	5.164	0.006
$NaGdF_4$:20%Eu^{3+}	4.892	0.006
$NaGdF_4$:25%Eu^{3+}	4.893	0.005
$NaGdF_4$:30%Eu^{3+}	4.533	0.005
$NaGdF_4$:50%Eu^{3+}	3.729	0.004

Inductively coupled plasma–optical emission spectroscopy (ICP−OES) measurements

The amount of Eu^{3+} in core, core-shell, and core-shell-shell NPs (mg/g of NPs) was determined via ICP−OES using Perkin-Elmer Optima 7000DV spectrometer. The NPs were dissolved in nitric acid (Rotipuran® Supra 69%, Carl Roth) and diluted to the required volume using DI water. The Eu^{3+} calibration solution was prepared by appropriately diluting the stock standard solution (single-element ICP standards, 1000 mg/L, Carl Roth).

Table S3. Amount of Eu^{3+} (mg/g of NPs) determined via ICP-OES.

**Sample**	**Core NPs**	**Core-shell NPs**	**Core-shell-shell NPs**
**Amount of Eu^{3+} (mg/g of NPs)**	41.50	26.36	8.01

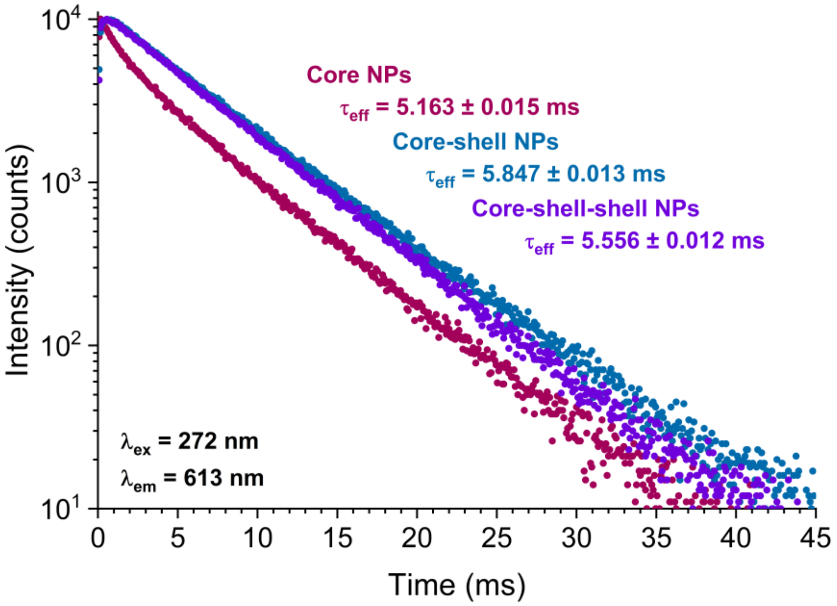

Figure S7. PL decay curves of Eu^{3+} (λ_{em} = 613 nm) in core, core-shell, and core-shell-shell NPs dispersed in acidified DI H_2O (adjusted with HCl, pH ca. 5.5). The concentration of Eu^{3+} in each sample was set to 20 mg/l.

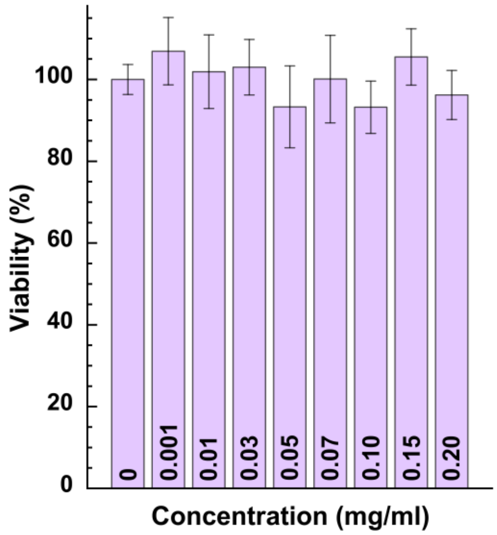

Figure S8. Viability (determined via XTT test) of human breast cancer cells MDA-MB-231 exposed to different concentrations of core-shell-shell NPs.
